# Establishment and characterization of a differentiated epithelial cell culture model derived from the porcine cervix uteri

**DOI:** 10.1186/1746-6148-8-31

**Published:** 2012-03-19

**Authors:** Katrin Miessen, Ralf Einspanier, Jennifer Schoen

**Affiliations:** 1 Freie Universität Berlin, Institute of Veterinary Biochemistry, Oertzenweg 19b, 14163 Berlin, Germany

## Abstract

**Background:**

Cervical uterine epithelial cells maintain a physiological and pathogen-free milieu in the female mammalian reproductive tract and are involved in sperm-epithelium interaction. Easily accessible, differentiated model systems of the cervical epithelium are not yet available to elucidate the underlying molecular mechanisms within these highly specialized cells. Therefore, the aim of the study was to establish a cell culture of the porcine cervical epithelium representing *in vivo*-like properties of the tissue.

**Results:**

We tested different isolation methods and culture conditions and validated purity of the cultured cells by immunohistochemistry against keratins. We could reproducibly culture pure epithelial cells from cervical tissue explants. Based on a morphology score and the WST-1 Proliferation Assay, we optimized the growth medium composition. Primary porcine cervical cells performed best in conditioned Ham's F-12, containing 10% FCS, EGF and insulin. After cultivation in an air-liquid interface for three weeks, the cells showed a discontinuously multilayered phenotype. Finally, differentiation was validated via immunohistochemistry against beta catenin. Mucopolysaccharide production could be shown via alcian blue staining.

**Conclusions:**

We provide the first suitable protocol to establish a differentiated porcine epithelial model of the cervix uteri, based on easily accessible cells using slaughterhouse material.

## Background

The uterine cervical epithelium protects the upper reproductive tract from insults providing a physical barrier, secretions containing bactericidal and virucidal agents and a pathogen-dependent direct immunomodulation [1-3]. During estrous, it takes part in direct sperm-epithelium interaction [4] as well as in the signal reception from seminal fluid [5].

To elucidate cell type-specific actions of hormones and cytokines, signal transduction pathways, cell-cell interactions, and gene expression in these highly specialized cells, model systems resembling the original tissue need to be developed.

Cervical cell cultures of a variety of species are already applied in various fields of science. They serve as *in vitro *systems for basic research [6], in oncological and microbiological studies [7-9] as well as for assessment of product-and pharmaco-toxicity [10,11].

The cells used in these studies are mainly derived from human ectocervical tissue, which *in vivo *is covered by a polarized, multilayered epithelium. However, the cells (primary, immortalized or transformed) are cultured as monolayers and therefore lost these tissue specific characteristics. Maintenance of multilayered growth and polarity is pivotal for the *in vivo*-like functionality of the ectocervical epithelium *in vitro*, as apical polarity forms physical paracellular and functional barriers based on cell-cell contacts [12-15].

Cell culture models used in basic research as well as in toxicology ideally should meet two requirements at the same time: to a) be easily and continuously available and b) resemble the *in vivo *properties of the specific cell type. Therefore, we investigated, if porcine material from the slaughterhouse could provide to establish a suitable and differentiated cell culture model of the uterine cervical epithelium. Pigs for slaughter are usually healthy and roughly of the same age. During the last decades the pig also became one of the favoured models for humans, since anatomy, physiology and genetics are highly comparable [16]. The mean length of estrous cycle and hormone profiles as well as cervical mucus production also resemble the human characteristics [7,17].

In order to provide a practical tool to analyze the complex pathways within the cervical epithelium, the aim of this study was to establish an accessible model of the porcine ectocervical epithelium based on tissue derived from slaughterhouse. Cell isolation and culture conditions were optimized in order to support proliferation and differentiation *in vitro*. The culture was characterized by specific markers to describe the cell type, state of differentiation and functionality in comparison to the native tissue.

## Results

### Cell transport and isolation

Transport conditions from the slaughterhouse to the laboratory (transportation time approximately 2 h) turned out to be a crucial factor for cell viability. Tissue that was transported in growth medium at room temperature showed best survival of the epithelial cells.

**Figure 1:**
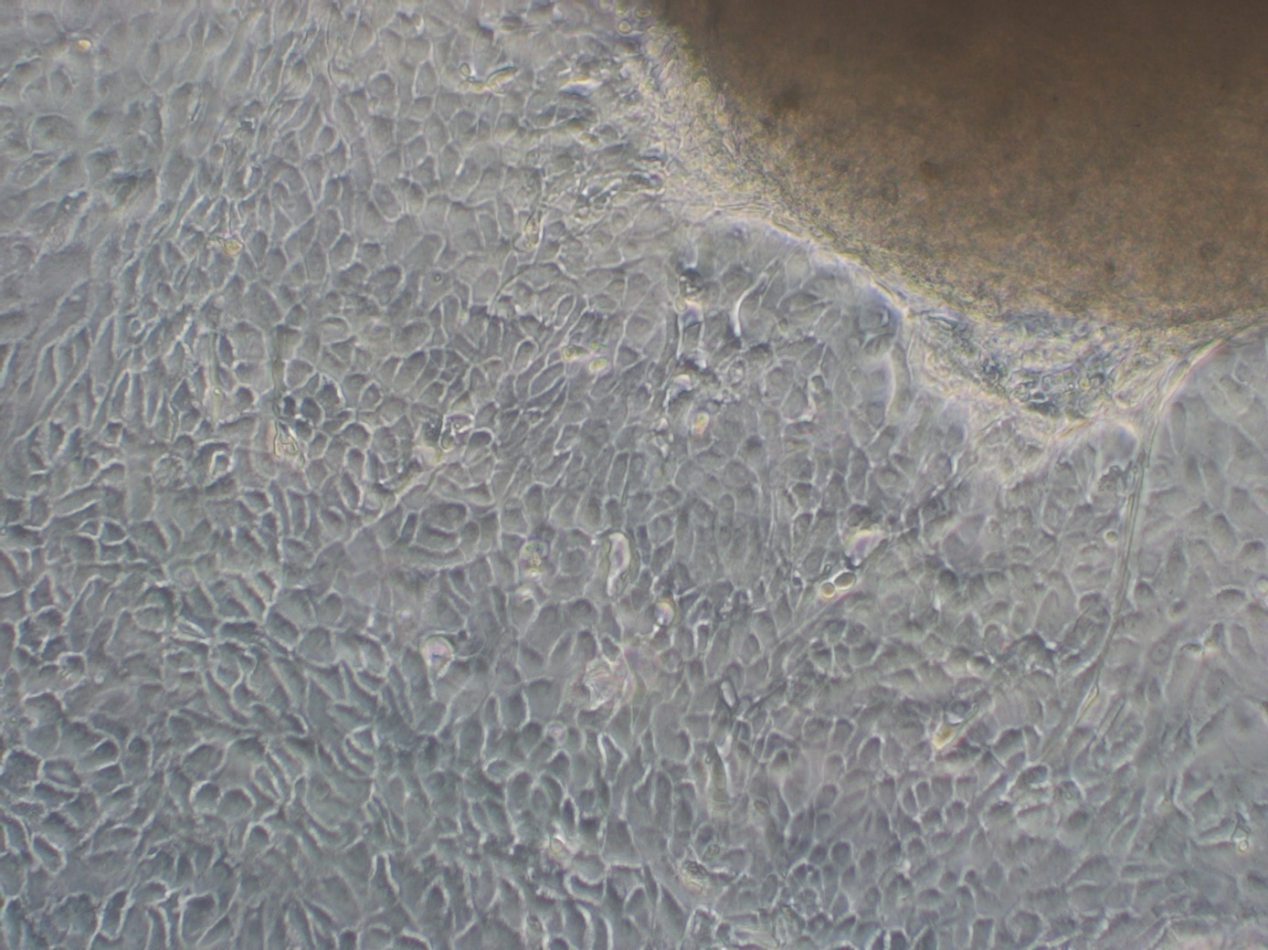
**Tissue explant from ectocervical tissue, cultured in Ham's F-12 containing 10% FCS for five days**. Epithelial cells grow out of the tissue and attach to the cell culture dish.

Pure epithelial cervical cells could reproducibly be isolated only by outgrowth from tissue explants (Figure 1) as other previously described cell isolation methods did not lead to a pure and viable primary cell population in our hands.

Primary cells can be passaged and cryopreserved using standard protocols.

**Figure 2:**
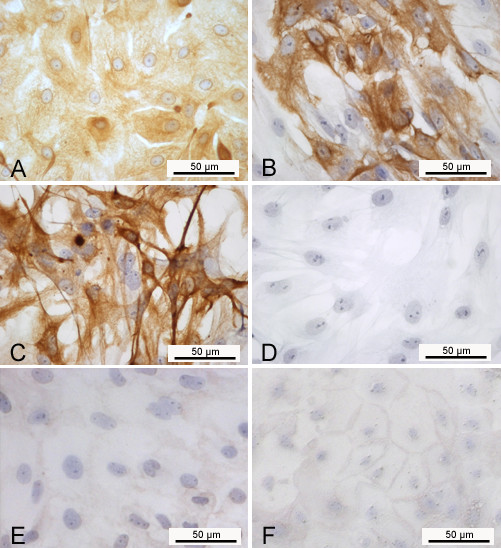
**Immunocytochemical detection of keratins and beta catenin in porcine cervical epithelial cells**. Keratins were detected in A) primary cells, B) passage one, C) passage 5; D) negative control. Beta catenin is shown in E) primary cells and F) passage one. Counterstain: hemalum.

**Figure 3:**
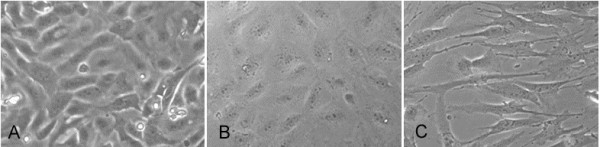
**Epithelial cells of the cervix uteri, conventionally cultured for 10 days in cell culture plates using Ham's F-12, containing FCS, EGF and insulin as growth medium**. A) primary cells, B and C) passage one. The passaged cells show an inhomogeneous morphology.

After passaging the cervical cells up to five times, the cultured cells were still viable and showed some epithelial characteristics (keratin staining). However, the epithelial morphology was completely lost in passage 5 (Figure 2). Already in passage one morphological alterations can be found in some parts of the monolayer (Figure 3).

### Initial media testing

Growth media were evaluated using a simple morphology score. The growth medium Ham's F-12 containing 10% FCS, antibiotics, antimycotics and antioxidants gained the highest score of 180 points concerning proliferation, the absence of vacuoles and epitheloid phenotype of the grown-out primary cells and cells in passage one (Table 1).

**Table 1:** Morphology scores of cervical epithelial cells grown in different growth media

Medium		Score
Ham's F-12	+10% FCS	180
DMEM/Ham's F-12 (1:1)	+10% FCS	170
Medium199	+10% FCS	165
DMEM	+10% FCS	165
McCoy's 5A	+10% FCS	140

The highest score achievable was 240 points per growth medium (8 points in three categories, 30 animals in total; primary cells after five days of culture: n = 15 animals; cells in passage one after two days of culture: n = 15 animals)

### Test of media conditioning and media additives by WST-1 assay

**Figure 4:**
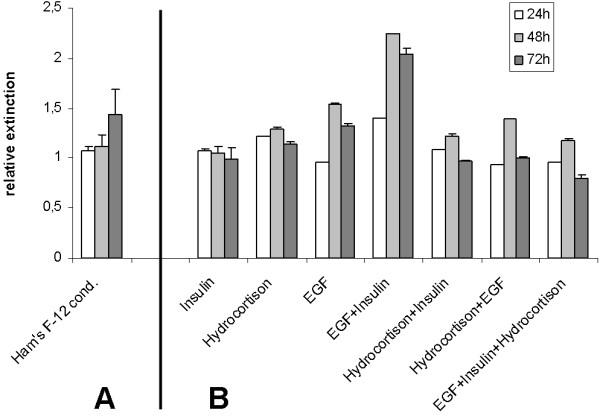
**Cervical epithelial cells of three animals were cultured for 24 h, 48 h and 72 h in different growth medium compositions**. Viability and proliferation were measured in triplicates using the Roche WST-1 Proliferation Assay. Extinctions of the media were measured at 450 nm after 30 min incubation with the WST-1 reagent. A) Ham's F-12 relative to the unconditioned medium, and B) conditioned Ham's F-12 with different additives relative to the basic conditioned Ham's F-12.

Conditioning of the growth medium and the addition of media supplements were investigated using cervical cells of passage one. Cells cultured in conditioned Ham's F-12 showed an increase in mitochondrial turnover of WST-1 of 12% after 48 h and 44% after 72 h relative to the unconditioned growth medium. Supplementation of EGF and porcine insulin to the conditioned Ham's F-12 led to a two-fold higher mitochondrial activity after 48 h and 72 h compared to the conditioned growth medium without any hormone substitution (Figure 4). Other additives or other additive combinations led to a less pronounced increase (EGF, EGF + hydrocortisone, EGF + hydrocortisone + insulin) or stagnation (insulin, hydrocortisone, insulin + hydrocortisone) of mitochondrial activity.

### Differentiated, multilayered cell culture of cervical cells

Primary cervical cells and cells of passage one were cultured on Millicell inserts either with access to growth medium from both basolateral and apical or in air-liquid interface with access to growth medium only from basolateral. We analyzed the respective setups histologically after three weeks considering *in vivo*-like morphology.

**Figure 5:**
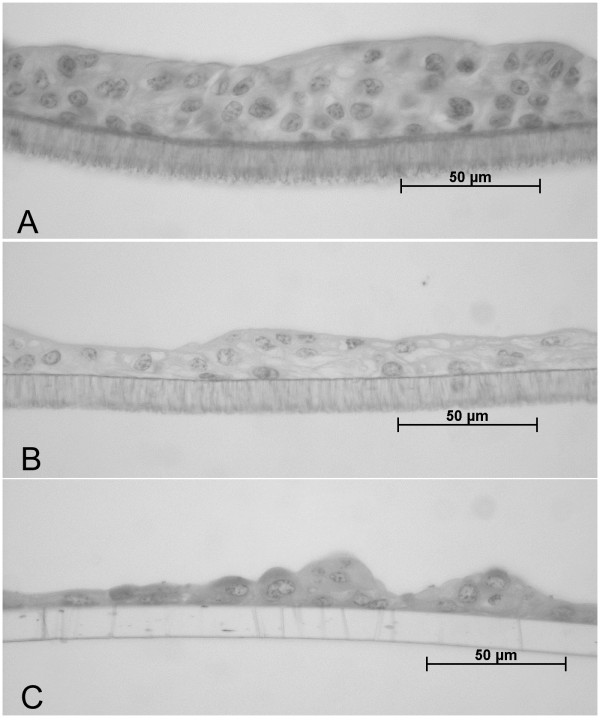
**Ectocervical epithelial cells cultured on hanging membranes with conditioned Ham's F-12 containing 10% FCS, EGF and insulin for three weeks**. A) Primary cells cultured in air-liquid interface (with access to growth medium only from basolateral), B) primary cells with access to growth medium from both basolateral and apical side, and C) epithelial cells of passage one cultured in air-liquid interface.

A differentiated phenotype was established when culturing primary cells in an air-liquid interface (without medium in the upper compartment) for three weeks. The cells showed a reproducible discontinuously stratified phenotype with up to six layers. When cultured conventionally with access to growth medium from both basolateral and apical the epithelial cells were mainly columnar and also showed partially multilayered growth. However, height of the multilayer was increased by growth in air liquid interface (conventional growth: up to 4 layers, air-liquid interface: up to six layers). After passaging, the epithelial cells grew as single-layer consisting of non-polarized cells. Passaged cells showed multilayered growth only in very few areas of the membranes (Figure 5).

### Characterization of the in vivo-like cell culture

**Figure 6:**
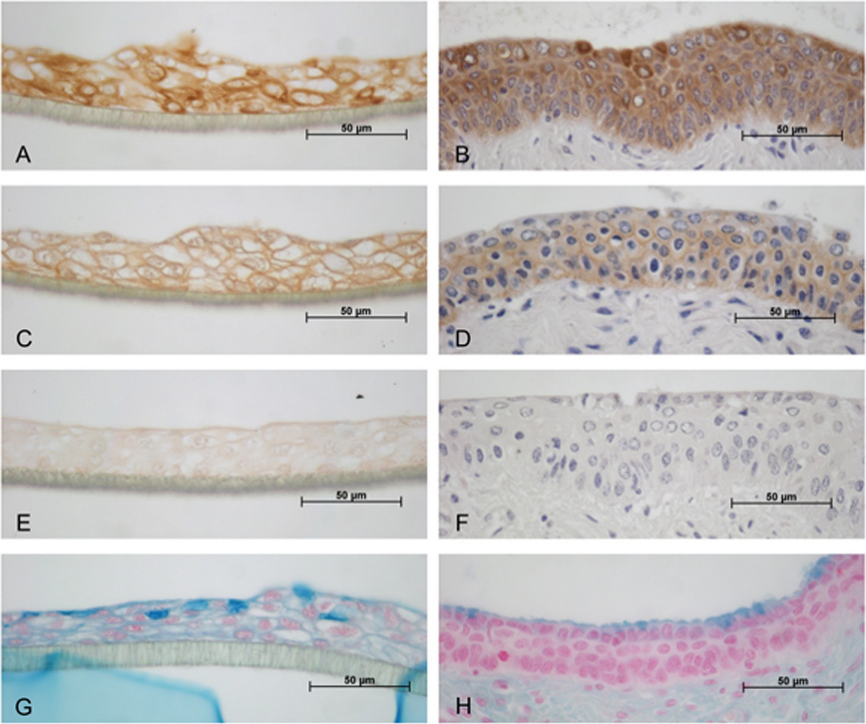
**Detection of keratins (A, B; E and F: negative control), beta catenin (C, D) and mucopolysaccharides (G, H) in porcine cervical epithelial cells**. Left column (A, C, E, G): primary cervical epithelial cells cultured in air-liquid interface for three weeks; Right column (B, D, F, H): ectocervical tissue sections.

Purity of the epithelial cell culture and differentiation was shown by immunohistochemistry using antibodies against keratin, as epithelial marker, and beta catenin, as part of adherens junctions. Cervical epithelial cells cultured on membranes in air-liquid interface for three weeks showed keratin-expression in the cytoplasm comparable to the ex vivo-epithelium (Figure 6A/B). Likewise, beta catenin could be visualized near the cellular membrane on cell culture and tissue sections (Figure 6C/D). In the tissue samples beta catenin expression is detectable in all layers of the porcine cervical epithelium, but the intensity of the immunostaining is lower in the superficial cell layer. This difference could not be detected in the *in vitro*-model.

To show cervix specific function mucopolysaccharides were visualised. The multilayered cell cultures showed a blue signal in the apical layers after alcian blue staining comparable to the *in vivo *situation (Figure 6G/H).

## Discussion

Epithelial cells of different tissues or species diverge substantially in their culture requirements. Thus, *in vivo*-like culture of epithelial cells necessitates optimization of the entire culturing process including transport, isolation, medium composition and culture conditions. In the present study we established a new protocol for a differentiated cell culture system of the porcine cervical epithelium, based on easily accessible slaughterhouse material. The morphology and tested functional markers of our culture system are comparable to the native tissue as shown by histology, immunohistochemistry and alcian blue staining. The use of fibroblast-conditioned medium supported proliferation of cervical epithelial monolayers suggesting that stromal growth factors or cytokines released into the medium are required for cell growth in these epithelia. The supplementation of the conditioned medium with EGF further optimized proliferation and mitochondrial activity of the cervical epithelial cells. Insulin intensified the proliferative effect of EGF, as cellular metabolic effects of EGF (next to synthesis of DNA, RNA and proteins) also include stimulation of glucose metabolism [18]. Providing an air-liquid interface to the primary cells promoted the formation of multilayers. In contrast to reports on cervical epithelial cells from other species [13,19] passaging the primary cells, however, caused disadvantageous effects. Already in passage one cells showed a decreased potential to form multilayers and extended passaging led to the loss of epitheloid cell character.

Cervical epithelial cells play a relevant role in nowadays research as cell cultures derived from this tissue are frequently used as *in vitro *models. The level of differentiation, however, varies widely between different culturing systems.

Cervical epithelial cells cultured as monolayers on solid support dedifferentiate [20] and are more susceptible to certain toxic agents than ex vivo tissue cultures or animal models [10,21]. Therefore, a fully differentiated three-dimensional *in vitro *system of the human cervicovaginal epithelium was recently established for toxicological studies [13,22]. This homologous system is surely ideal for human research, but the general availability and the resulting small number of possible biological replicates are limiting factors for its usability. Advanced cell culture technologies like the rotating wall vessel bioreactor showed to provide differentiated vaginal epithelial cells from immortalized cells [23]. However, special equipment is needed to conduct this technique.

The porcine cells used for our model of the cervical epithelium are unlimitedly accessible and maintenance as well as handling of the cells can be carried out in a standard cell culture lab. The differentiated cell culture resembles physiological properties of the native tissue and can, therefore, be called *in vivo*-like model.

However, with regard to the different scientific questions addressable with this model system (pathogen-dependent immunomodulation, sperm-epithelium interaction, signal reception etc.) we did not carry out a functional characterization of the cell culture. It is now essential to describe and validate its specific functional properties by focusing directly on the particular processes under investigation.

## Conclusions

We developed an *in vitro *system modelling the mammalian cervical epithelium that is cost-effective and easily available as it is based on slaughterhouse by-products. It is a practical tool in veterinary science and might also promote human reproductive research.

## Methods

### Overview

We tested different methods to isolate epithelial cells from porcine cervical tissue. Standard growth media and a range of additive-compositions were investigated regarding their influence on proliferation and morphology. The cultured cells were characterized by histology, immunocyto-and-histochemistry. Studying differentiation, we cultured tissue explants and cells from passage one on hanging membranes either with access to medium from both basolateral and apical side or in an air-liquid interface with access to medium only from the basolateral side.

### Material

Media, antibiotics and serum for cell culture were supplied by Biochrom AG, Berlin, Germany. All other chemicals were obtained from Sigma-Aldrich, St. Louis, USA, unless otherwise indicated.

### Tissue preparation and transport conditions

Porcine uterine cervices were collected in a local slaughterhouse from healthy, 6-month-old, pre-pubertal animals, approximately 15 min after death. Ovaries of all animals only showed small follicles of the same size as a sign for cyclic inactivity. The organs were washed immediately with Dulbecco's PBS.

Conditions of transport were investigated concerning the influence on cell viability after isolation. Cervical tissues were transported to the laboratory either dry or in growth medium containing 10% FCS, fetal calf serum (on ice or at room temperature).

After optimization of transport conditions the cervical tissues were transported in basic growth medium (Medium 199, or after intial media testing: Ham's F-12) containing 10% FCS at room temperature within 2 hours.

For histology, tissues of four animals were transversally cut in 5 mm thick pieces and fixed in chilled Bouin's solution on-site.

### Isolation of cervical epithelial cells

To isolate pure epithelial cells from porcine cervical tissue, we applied different previously published enzymatic digestion methods [7,13,24] as well as a modified protocol for cellular outgrowth from primary tissue explants [25]. Briefly, cervices were opened longitudinally and rinsed with Dulbecco's PBS. Tissue pieces of the ectocervical mucosa (approximate length 1 cm, width 2 mm and height 1 mm) were cut with scissors. 10 pieces were placed (mucosal side up) in a 25 cm^2 ^culture flask with 2 mL of growth medium. After initial media testing, the growth medium consisted of conditioned Ham's F-12, 10% FCS, 10 ng/mL murine EGF (epidermal growth factor), 1 μg/mL porcine insulin, 100 U/mL penicillin, 100 μg/mL streptomycin, 50 μg/mL gentamycin, 1 μg/mL amphotericin B, 10 μg/mL reduced glutathione and 10 μg/mL ascorbic acid as antioxidants. After 48 and 96 h, culture medium was changed. After five days the tissue explants were removed from the culture vessel.

If necessary for further experiments, the cells were passaged (3 mL of Accutase, 20 min incubation at 37°C) after five days of culture.

Cells were cryopreserved using a standard protocol. 5 × 10^4 ^cells were resuspended in 500 μL CryoMaxx (PAA Laboratories GmbH, Pasching, Austria) and cooled down to -80°C (1°C/min) before storage in liquid nitrogen.

### Immunocytochemistry

To proof cell type and purity of the collected cells we applied immunocytochemistry against pan-keratins. Differentiation was shown using an antibody against beta catenin, as part of adhearens junctions. For primary antibodies and protocol details see Table 2.

**Table 2:** Antibodies and protocol details for immunohistochemistry (IHC) and immunocytochemistry (ICC) procedures

	name	manufacturer	dilution	blocking solution
**IHC**	Beta Catenin, ab6302	Abcam plc, Cambridge, Uk	1:160.000 in PBS	no block
	Anti human cytokeratin, clones AE1/AE3	DakoCytomation, Glostrup, Denmark	2.4 μg/mL in PBS	Casein, Candor Bioscience, Münster, Germany
**ICC**	Beta Catenin, ab6302	Abcam plc, Cambridge, Uk	1:2.000 in PBS	no block
	Anti human cytokeratin, clones AE1/AE3	DakoCytomation, Glostrup, Denmark	1 μg/mL in PBS	no block

Either tissue explants or 1 × 10^5 ^cervical cells in passage one were grown on Superfrost Plus^® ^slides (Gerhard Menzel, Glasbearbeitungswerk GmbH & Co. KG, Braunschweig, Germany) until confluence. They were rinsed with Dulbecco's PBS, fixed in ice-cold acetone for 10 min and washed three times in Dulbecco's PBS. Blocking of unspecific binding sites was not necessary. The primary antibody was incubated for 1 h at room temperature (pan-keratins) or at 4°C overnight (beta catenin). Mouse immunoglobulin G fraction or normal rabbit serum (DakoCytomation, Glostrup, Denmark) served as negative control. After three washing steps in PBS-T 0.01%, the secondary antibody (Goat Anti-Rabbit or Anti-Mouse Poly-HRP, Thermo Scientific, Rockford, USA) was incubated for 30 min at room temperature. After three more washing steps, diaminobenzidin (DAB-/Metal Concentrate 10×, Thermo Scientific) was used as chromogen.

### Initial media testing

To evaluate different media for further use as basic growth medium, we applied a morphology score.

Cervical epithelial cells of 30 animals were cultured (in conventional cell culture plates) as described above in five different growth media containing 10% FCS as well as antibiotic, antimycotic and antioxidant reagents (Table 3). Cell morphology of primary (five days after explantation) and cells in passage one (two days after passaging) was assessed by light microscopy. The morphology was scored concerning proliferation, presence of vacuoles and epitheloid phenotype (Table 4). The highest achievable score of each medium was 240 points (8 points in three categories, 30 animals in total; primary cells after five days of culture: n = 15 animals; cells in passage one after two days of culture: n = 15 animals).

**Table 3:** Media composition in the different steps of cell culture optimization

Initial media testing	*Basic media*	*Supplementation*
	DMEM +10% FCS	-
	DMEM/Ham's F-12 (1:1) +10% FCS	-
	Ham's F-12 +10% FCS	-
	McCoys-5A +10% FCS	-
	Medium 199 +10% FCS	-
**Media conditioning and additives**	*Basic media*	*Supplementation*
	Ham's F-12 +10% FCS	-
	Ham's F-12 +10% FCS, cond.	-
	Ham's F-12 +10% FCS, cond.	+ EGF
	Ham's F-12 +10% FCS, cond.	+ insulin
	Ham's F-12 +10% FCS, cond.	+ hydrocortisone
	Ham's F-12 +10% FCS, cond.	+ EGF, insulin
	Ham's F-12 +10% FCS, cond.	+ insulin, hydrocortisone
	Ham's F-12 +10% FCS, cond.	+ EGF, hydrocortisone
	Ham's F-12 +10% FCS, cond.	+ EGF, insulin, hydrocortisone
**Differentiated cell culture**	*Lower compartement*	*Upper compartment*
	Ham's F-12 +10%FCS, cond., EGF, insulin	a) Ham's F-12 +10%FCS, cond., EGF, insulin
		b) no medium (air-liquid interface)

*DMEM *Dulbecco's MEM medium, *FCS *fetal calf serum, *cond*. conditioned. Concentration of the supplements: 10 ng/mL murine EGF, 1 μg/mL porcine insulin, 0.5 μg/mL hydrocortisone. All growth media were supplemented with 100 U/mL penicillin/100 μg/mL streptomycin, 50 μg/mL gentamycin, 1 μg/mL amphotericin B, 10 μg/mL reduced glutathione and 10 μg/mL ascorbic acid.

**Table 4:** Morphology score criteria applied in the initial media testing

score	Proliferation (P0)	Proliferation (P1)	Vacuole formation	Phenotype
3	Continuous margin of cells around explants	> 90% confluence	no vacuoles	-
2	Discontinuous margin of cells around explants	70%-90% confluence	< 10% cells vacuolated	epitheloid phenotype
1	single cells and small clusters of cells around explants	< 70% confluence	> 10% cells vacuolated	-
0	not assessable, all or many cells dead	not assessable, all or many cells dead	not assessable, all or many cells dead	fibroblastoid phenotype

*P0 *primary cells, *P1 *cells in passage one

### Preparation of conditioned medium

The growth medium with the best results in the initial media testing was further modified. Conditioned growth medium (mimicking stromal-epithelial interaction) was prepared referring to standard protocols [19]. In short, 5 × 10^5 ^3T3 Swiss albino embryo fibroblast cells (ATCC, Manassas, USA) were grown in a 75 cm^2 ^cell culture flask in 20 mL Ham's F-12, 10% FCS. After 48 h (subconfluence), fresh growth medium was incubated for 24 h on the fibroblasts. The sterile filtered supernatant was stored at -20°C. To prepare conditioned growth medium, one part of the supernatant was diluted in two parts of unconditioned medium.

### Test of media conditioning and media additives by WST-1 assay

Influence on cell viability and proliferation of different growth media (for media composition details see Table 3) was monitored using WST-1 Proliferation Assay (Roche Deutschland Holding GmbH, Grenzach-Wyhlen, Germany) according to the manufacturer's guide. In short, 1 × 10^4 ^cervical cells of passage one were seeded in 96-well culture dishes. 24 h, 48 h and 72 h after seeding, cells were incubated for 30 min with the reagent and WST-1 turnover was measured photometrically at wavelength 450 nm and a reference wavelength of 630 nm. All measurements were conducted for three animals as triplicates.

### Differentiated cell culture

Cells were grown on permeable membranes (6-well and 24-well Millicell hanging inserts, PET membrane, Millipore, Temocura, Canada) hanging in tissue culture plates and creating two compartments, a basolateral and an apical one. We applied different cultivation setups: access to complete growth medium from either sides or air-liquid interface for nutrition exclusively from the basolateral side.

1 × 10^5 ^cervical epithelial cells (passage one) were seeded in 24-well inserts containing 200 μL growth medium with 1 mL medium in the well. To culture non-passaged cells on membranes, four pieces of cervical mucosa were placed in a 6-well insert containing 500 μL growth medium with 2 mL medium in the well. The culture plates were incubated at 37°C, 5% CO2 in a humid chamber. After 48 and 96 h growth medium was changed. After five days of culture, the mucosal pieces were carefully stripped off the membrane. To achieve an air-liquid interface, growth medium was completely removed from the insert. After three weeks of culture, the membranes were carefully drawn off the plastic inserts and further processed for histology.

### Preparing ex vivo tissue and cell culture membranes for histology

For immunohistochemistry, the cervical tissue or cells on membranes (embedded in 1.2% agarose to ensure a cutting ancle of 90°) were fixed in Bouin's solution at 4°C for at least 24 h. After washing in 4% buffered formalin, the samples were dehydrated and embedded in paraffin. Sections of approximately 5 μm were cut, mounted on Superfrost Plus^® ^slides and dried at 37°C overnight.

Hemalum/eosin staining to identify cellular morphology and alcian blue staining at pH 2.5 to visualize mucus production was performed for both tissue and membrane sections.

### Immunohistochemistry

To proof the differentiation state of the epithelial cells cultured on membranes, we applied immunohistochemistry using antibodies against keratins and beta catenin (for primary antibodies and protocol details see Table 2).

After deparaffination and rehydratation, sections were washed in Dulbecco's PBS. Antigens were demasked by boiling slides for 2 min and cooling down for 20 min in citrate buffer, pH6. Endogenous peroxidase was blocked in 3% H_2_O_2 _in methanol (twice, 15 min). Sections were incubated with blocking solution at 37°C in a humid chamber for 1 h. Sections were incubated with primary antibodies at 4°C in humid chamber over night. Mouse immunoglobulin G fraction or normal rabbit serum respectively (DakoCytomation) served as negative control. After washing in PBS-T 0.01% (3 times, 15 min), slides were incubated with goat anti-mouse or anti-rabbit secondary antibody (Thermo scientific) conjugated with horseradish-peroxidase for 30 min. Peroxidase was visualized using diaminobenzidin.

## Authors' contributions

KM participated in study design, carried out tissue collection, cell culture experiments, histological sample procedures, immunohistochemistry and other histological studies, and drafted the manuscript; RE participated in study design and supervision, and revised the manuscript; JS conceived of the study, coordinated and supervised the study performance, and drafted and revised the manuscript. All authors read and approved the final manuscript.
